# Hyperimmune Globulins for the Management of Infectious Diseases

**DOI:** 10.3390/v15071543

**Published:** 2023-07-13

**Authors:** Ilaria Pati, Mario Cruciani, Fabio Candura, Maria Simona Massari, Vanessa Piccinini, Francesca Masiello, Samantha Profili, Lucia De Fulvio, Simonetta Pupella, Vincenzo De Angelis

**Affiliations:** National Blood Centre, Italian National Institute of Health, 00161 Rome, Italy; crucianimario@virgilio.it (M.C.); fabio.candura@iss.it (F.C.); mariasimona.massari@iss.it (M.S.M.); vanessa.piccinini@iss.it (V.P.); francesca.masiello@iss.it (F.M.); samantha.profili@iss.it (S.P.); lucia.defulvio@iss.it (L.D.F.); simonetta.pupella@iss.it (S.P.); vincenzo.deangelis@iss.it (V.D.A.)

**Keywords:** hyperimmune globulins, infectious diseases, human immunoglobulin, therapy, convalescent plasma

## Abstract

This review is focused on the use of hyperimmune globulin therapy to treat some infectious diseases of viral or bacterial origin. Despite the introduction of antibiotics and vaccines, plasma immunoglobulin therapy from whole blood donation can still play a key role. These treatments provide passive transfer of high-titer antibodies that either reduces the risk or the severity of the infection and offer immediate but short-term protection against specific diseases. Antibody preparations derived from immunized human donors are commonly used for the prophylaxis and treatment of rabies, hepatitis A and B viruses, varicella-zoster virus, and pneumonia caused by respiratory syncytial virus, *Clostridium tetani*, *Clostridium botulinum*. The use of hyperimmune globulin therapy is a promising challenge, especially for the treatment of emerging viral infections for which there are no specific therapies or licensed vaccines.

## 1. Introduction

Passive antibody therapy has been used for the prevention and treatment of many microorganisms responsible for infections (viruses, bacteria, fungi, and protozoa) [1,2]. Before the availability of antibiotics, antibodies were the only specific treatment for certain infections [3]. Although antibiotics and vaccines have largely replaced their role in the treatment of infections, passive antibody administration (serum therapy) is still important in the field of infectious diseases due to the increase in antimicrobial resistance as well as the emergence of new pathogens [1,2,3,4]. Passive antibody therapy can also bypass critical situations related to immune response deficiency [3].

Antibody-based therapies were the primary measure to treat many infectious diseases, at the beginning (late nineteenth century) as human or animal plasma or serum, then as pooled human immunoglobulin (IG) for intravenous (IVIG) or intramuscular/subcutaneous (IM/SCIG) use, or as hyperimmune globulins (HIGs, high-titer human IVIGs or IGs from immunized or convalescent donors), and more recently as monoclonal antibodies (mAbs) [3,4]. The use of antitoxins obtained from animals was limited by safety profile [5]. Currently, heterologous serums have been replaced by other drugs, such as antibiotics, vaccines, or HIGs from healthy donors [5,6].

IG formulations are sterile, nonpyrogenic preparations of globulins containing many antibodies normally present in adult human blood. IGs are produced from plasma obtained from whole blood donation as recovered plasma or from plasmapheresis.

IVIG is a highly purified product consisting mostly of immunoglobulin G (IgG) with a half-life of 21–28 days [7]. HIG products are manufactured from donors with high Ig titers with specificity to antigenic determinant(s) of interest. High titers of these donors can be achieved through natural immunity, prophylactic immunizations, or through targeted immunizations. HIG products should contain at least fivefold-increased titers compared to standard preparations of IVIG [7].

Despite improvements in antimicrobial therapies, there are a large number of pathogens that remain difficult to treat and others for which no specific chemotherapy exists. Hence, polyclonal immunoglobulin continues to be used for the treatment of a variety of infectious diseases and infection-related disorders, often based on anecdotal and off-label experience [8]. The HIGs may also be used in combination with vaccines to confer temporary immunity, as vaccines typically need time to produce protective immunity.

There are limited conditions for which IVIG therapy is approved by the US Food and Drug Administration (FDA) and the European Medical Agency (EMA) [4,5,8,9,10]. These conditions include both infectious and non-infectious states, such as idiopathic thrombocytopenia purpura, Kawasaki disease, primary immunodeficiency, secondary hypogammaglobulinemia caused by B-cell chronic lymphocytic leukemia, prophylaxis in stem cell transplant recipients, and prophylaxis in pediatric HIV/AIDS. The cumulative evidence, along with the cost-effectiveness, must be taken into account when considering IG therapy in the contest of a limited supply of plasma for fractionation as faced during the pandemic years and nowadays not yet recovered.

While polyclonal IG products are nonspecific in their composition, hyperimmune products have an augmented concentration of antigen-specific antibodies, and they have been and are still being used with success in preventing viral or bacterial infections. These treatments provide passive transfer of high-titer antibodies that either reduces risk or reduces the severity of infection. HIG provides immediate but short-term protection against specific diseases.

The use of passive immunotherapy, also through the use of HIGs, is a promising challenge, especially for the treatment of emerging viral infections for which, to date, there are no specific antiviral therapies or licensed vaccines. For example, convalescent plasma (CP) from convalescent donors has been successfully used for empirical treatment and post-exposure prophylaxis during outbreaks of Ebola virus disease [11,12,13].

## 2. Current Role of Hyperimmune Globulins for the Management of Infectious Diseases

Hyperimmune globulins continue to have an important role in the treatment of infectious diseases, as also shown by the demand for specific IGs in Italy from 2017 and 2021. Table 1 shows these data, produced by the National Blood Centre, on the basis of the elaborations of traceability information flow provided by the Italian Ministry of Health every year. The use of HIG shows fluctuations in demand over this five-year period that are more or less significant and diversified depending on the type of IGs used. Particularly significant is the use of tetanus IG which remains broadly stable, standing around 2 IU per capita.

The main indications for passive immunotherapy are summarized in Table 2.

## 3. Antibody Preparations

### 3.1. Tetanus

Since the 1960s, a human tetanus immune globulin (TIG) has been available. In countries where human TIG is not available, equine antitoxin is still used [3]. Human polyvalent IVIG can also be used, but it contains variable levels of tetanus antitoxin. TIG is prepared from the plasma of donors immunized with tetanus toxoid; donors most suitable for boosting appear to be males under 25 years old with a tetanus antibody level of around 8 IU/mL prior to boosting [14]. TIG contains tetanus antitoxin antibodies, which neutralize the free form of the toxin produced by Clostridium tetani, but not the toxin bound to nerve endings [7]. At present, the Centers for Disease Control (CDC) recommend 1 dose of 250 IU TIG intramuscularly in susceptible persons who suffer high-risk wounds, in combination with a tetanus toxoid-containing vaccine for the development of long-term immunity [16]. Similar treatment is recommended for established tetanus infection; the usually recommended dose of TIG is from 3000 to 6000 IU, although some experts recommend a dose of 500 international units (IU), which appears to be as effective as higher doses [3,16,17].

Results of a meta-analysis of clinical trials show that intrathecal administration of equine antitoxin serum (ATS) or TIG is more beneficial than intramuscular administration in the treatment of tetanus [18].

### 3.2. Clostridium difficile

*C. difficile* is responsible for antibiotic-associated diarrhea and pseudomembranous colitis. The disease can be severe, particularly in immunocompromised subjects, and is usually treated with oral antibiotics (metronidazole, vancomycin, or fidaxomicin). The incidence of treatment failure of *C. difficile*-associated diarrhea following metronidazole treatment has increased, particularly among specific patient subsets, including elderly and immunocompromised patients [5]. Immunoglobulin therapy for *C. difficile* infection has been used, but the results are inconsistent [5]. More recently, the mAb bezlotoxumab has been introduced as an adjunctive treatment to reduce the risk of recurrence of *C. difficile* infection in adult patients [7]. The results of a recent systematic review demonstrated the effectiveness and safety of bezlotoxumab for the prevention of recurrent *C. difficile* infection and that its use may be a valuable therapeutic option for severe *C. difficile* infection rather than mild cases [19].

### 3.3. Botulism

Food-borne and wound botulism can be treated with equine antitoxin [3,5]. Equine antitoxin is not indicated in the treatment of infant botulism, while the results of a randomized controlled trial (RCT) show that prompt treatment of infant botulism type A or type B with hyperimmune globulin (BIG-IV) was safe and effective in shortening the length and cost of the hospital stay and the severity of illness [20]. A Cochrane review supports the use of BIG-IV for infant intestinal botulism and shows that adverse events were probably no more frequent with IG than with placebo; no evidence about the use of other medical treatments, including serum trivalent botulism antitoxin, was found [21].

### 3.4. Diphtheria

Diphtheria is under control in industrialized countries. Right now, no human diphtheria IG product is available. Treatment of Corynebacterium diphtheriae infection relies on an equine antitoxin in conjunction with antimicrobial agents [3,5]. Due to the risk of allergic reactions, post-exposure prophylaxis with antitoxin is not recommended, but rather vaccination and antimicrobial therapy should be implemented.

### 3.5. Cytomegalovirus (CMV)

CMV is a common infection occurring in patients undergoing solid organ transplantation (SOT) or hematopoietic stem cell transplantation (HSCT). CMV-specific hyperimmunoglobulin (CMVIG) is prepared from donors with high anti-CMV titers, but regular IVIG also contains CMV antibodies at lower titers [8]. CMVIG and regular IVIG has been used extensively, either prophylactically or pre-emptively, in SOT and HSCT, and the results of clinical trials have been the object of systematic reviews and meta-analysis [22,23,24]. A Cochrane review [22] concluded that in patients undergoing HSCT, routine prophylaxis with IVIG is not supported, but its use may be considered in lymphoproliferative disorder patients with hypogammaglobulinemia and recurrent infections for a reduction in clinically documented infections. Further clinical trials on the use of adjunctive immunoglobulin products in HSCT recipients with CMV disease are required, considering the high mortality of this disease and the tolerability of IG products. Prophylactic administration of CMVIG after solid organ transplantation was associated with beneficial effects in two systematic reviews [22,23]. In one of these reviews [22], CMVIG was associated with improved total survival, reduced CMV disease, and CMV-associated deaths. The other review [24] concluded that prophylactic CMVIG treatment in patients undergoing solid organ transplantation may be beneficial in preventing CMV infection, particularly in those at high risk of CMV infection or disease.

The efficacy and safety of CMVIG have also been evaluated for the prevention of the vertical transmission of congenital CMV (CCMV). The results of a meta-analysis showed a promising efficacy of hyperimmunoglobulin therapy among pregnant women with confirmed primary infection, which disappeared when secondary infection was included.

The efficacy and safety of HIG have been demonstrated in clinical trials and meta-analyses [25,26,27]. Many observational and controlled trials have shown that CMV-specific HIG is safe and can significantly decrease maternal–fetal transmission of CMV infection and, mostly, the occurrence of CMV disease.

### 3.6. Hepatitis A (HAV)

IVIG has been used extensively for the prevention of HAV, both as post-exposure prophylaxis or prophylaxis in travelers to endemic areas [3]. IVIG prevents early clinical disease, while subclinical HAV viremia stimulates the development of a long-lasting antibody response [3]. The usual post-exposure dose of IG is 0.02 mL/kg given promptly after exposure and no later than 2 weeks [28]. Infants born to hepatitis-A-infected mothers should also receive IG. The use of IVIG has decreased since the introduction of HAV vaccines [3]. Highly effective HAV vaccines are available for pre-exposure prophylaxis, and this has reduced the pre-exposure use of IVIG for high-risk individuals who could not be vaccinated, individuals with an allergy to vaccine components, or travelers declining vaccination [5,29,30]. Likewise, HAV vaccination is now also used for post-exposure prophylaxis since it offers equivalent protection to IVIG when administered within 2 weeks of HAV exposure [31]. Moreover, the prevalence of HAV antibodies in developed countries is progressively declining, making concerns about the antibody levels in IG preparation justified [32,33].

### 3.7. Hepatitis B (HBV)

HBV immunoglobulin (HBIG) from donors with high anti-HBsAg titers is a highly effective agent in post-exposure prophylaxis of mother-to-child transmission and post-transplant treatment of HBV [3]. HBIG is 80 to 90% effective in preventing infection when given within 24 h of exposure (e.g., needle stick, sexual exposure, or mucous membrane exposure) [34]. The usual HBIG dose is 0.06 mL/kg. HBIG offers prompt but short-lived protection against infection with HBV, and an HBV vaccine should also be started after acute exposure for long-lasting protection. HBIG (0.5 mL) can be administered to the infant either alone or in combination with HBV vaccine [35]. However, in a minority of cases, transmission may also occur during the prepartum period and is not preventable by the prompt post-partum use of HBIG and vaccine.

Patients with HBV who undergo liver transplantation are at increased risk of HBV recurrence. The introduction of treatment with HBIG during the 1990s and later the incorporation of oral antiviral drugs has improved the prognosis of patients with liver transplantation due to HBV [36]. There are various treatment strategies for preventing HBV recurrence in liver transplant patients. Generally, these regimens include oral nucleoside/nucleotide analogs, HBIG, and vaccines or a combination of these drugs. The treatment strategy of choice should be based on cost-effectiveness, along with other patients underlying conditions. In this setting, studies indicate that the new, more potent oral antiviral agents (e.g., entecavir, tenofovir) are more cost-effective than HBIG in most cases [37].

### 3.8. Respiratory Syncytial Virus (RSV)

RSV infections are frequent causes of mild to severe respiratory illnesses, often requiring hospitalization and occasionally responsible for death in infancy and early childhood. Human polyclonal RSV-specific IgG antibodies (RSVIG) and RSV-specific monoclonal antibodies have been investigated in several clinical trials for the prevention, amelioration, or treatment of RSV infections [38].

A Cochrane review assessed the effects of IG and mAb for the treatment of RSV-proven lower respiratory tract infections in children aged up to three years admitted to the hospital [39]. All trials were conducted in high-income countries, and data from populations in which the rate of death from RSV infection is higher are lacking. For hospitalized infants with RSV infection, low-certainty evidence suggests that IG and mAb (motavizumab or palivizumab) may result in unimportant absolute differences in mortality and few to no differences in length of hospitalization, need for and duration of mechanical ventilation, need for and duration of supplemental oxygen, need for intensive care unit (ICU) admission, or any adverse events. However, the number of participants included in most analyses was insufficient to yield reliable results.

For infants (mostly at high risk of RSV owing to prematurity or comorbidities), prophylaxis with palivizumab reduces hospitalization due to RSV infection and results in little to no difference in mortality or adverse events [40]. Moreover, palivizumab results in a slight reduction in hospitalization due to respiratory-related illness and may result in a large reduction in RSV infections. Palivizumab also reduces the number of wheezing days. These results may be applicable to children with a high risk of RSV infection due to comorbidities.

Palivizumab [41] is preferred to RSV-IVIG because of its lower cost and its ease of administration (intramuscular versus intravenous for RSV-IGIV) [3]. The most recent American Academy of Pediatrics (AAP) guidelines recommend palivizumab prophylaxis with a maximum of five monthly doses only in the first year of life for otherwise healthy infants born before 29 weeks gestation and for infants born before 32 weeks gestation with chronic lung disease of prematurity (CLD) defined as a requirement for supplemental oxygen for at least 28 days after birth [42]. Prophylaxis is no longer recommended in the second year of life, except for infants with CLD still requiring oxygen, corticosteroids, or diuretics.

### 3.9. Varicella-Zoster Virus (VZV)

Varicella-zoster IG (VZIG) is prepared from the plasma of patients recovering from varicella-zoster with high titers of VZIG antibodies [9]. When given shortly after exposure, VZIG can prevent or modify varicella (3). VZIG is used for the prevention of VZV in susceptible immunodeficient or immunosuppressed children exposed to chickenpox or shingles. It is also used in susceptible women during late pregnancy, in newborns whose mother develops chickenpox perinatally, and in exposed premature infants of less than 28 weeks gestation. It is not of benefit in established chickenpox or zoster infection [43]. Due to the availability of acyclovir, VZIG is not indicated for healthy exposed adults [3].

After reviews of the effectiveness of antivirals and varicella zoster immunoglobulin (VZIG) in the prevention of chickenpox, antiviral medication is now the post-exposure treatment of choice for all immunosuppressed patients and pregnant women, regardless of stage in pregnancy [44]. VZIG will only be issued for susceptible neonates exposed within one week of birth (either in utero from maternal infection or post-delivery) or if oral antivirals are contraindicated due to malabsorption or renal toxicity or because the patient is less than 4 weeks of age.

Global supplies of a range of IG products, including VZIG, have been limited in recent years, and supplies are likely to remain constrained in the future [44]; hence, the ability to identify suitable alternative products not derived from blood is important.

### 3.10. Rabies

Rabies is an essentially fatal disease that is preventable with the timely administration of post-exposure prophylaxis (PEP). PEP of rabies requires both vaccine and IG administration in addition to wound cleansing [3]. Current recommendations are to use human rabies immunoglobulin (HRIG) for up to 7 days after exposure if it is not given immediately. If anatomically feasible, HRIG should be fully administered in and around the wound, and the first vaccine dose should be given at an anatomically distant location to prevent neutralization by HRIG [5]. HRIG provides immediate, temporary rabies virus-neutralizing antibodies until the patient responds to active immunization and produces virus-neutralizing antibodies [7]. Human monoclonal antibodies for the rabies virus have been developed [45,46]. A recombinant human monoclonal antibody (SII RMAb) was tested in a PEP regimen in comparison with an HRIG-containing PEP regimen [47]. The PEP regimen containing the mAb was safe and demonstrated noninferiority to HRIG PEP in rabies virus-neutralizing activity production. This novel mAb potentially offers a safe and potent alternative for the passive component of PEP and could significantly improve the management of bites from suspected rabid animals.

### 3.11. SARS-CoV-2

Antibody therapies such as CP, IG, and monoclonal antibodies have emerged as major potential therapeutics for coronavirus disease 2019 (COVID-19) [48,49]. The efficacy of antibody therapies requires the administration of specific antibodies, given early in the course of the disease and in sufficient amounts [50]. IGs have been administered as an immunosuppressive treatment in late COVID-19 stages or as an antiviral treatment in pre-exposure prophylaxis, post-exposure prophylaxis, and treatment of early COVID-19 stages. As immunosuppressants in the late COVID-19 stages, results of clinical trials and meta-analysis indicate a lack of efficacy [51]. Human-neutralizing IG (HIG) has been traditionally derived from selected CP donors. HIG from pooled CP has been developed [52,53], but further clinical trials are needed to define the safety and effectiveness of passive immunization treatment options for COVID-19. To date, COVID-19 HIG has been shown to retain neutralizing potency against past variants of concern (e.g., Alpha, Beta, Gamma, Delta/Delta+, Eta, Iota, Kappa, Lambda, Mu), but no data are available for Omicron [48]. CP and IG (either standard IG or HIG) have different manufacturing times, which makes CP preferable early in pandemics, while IGs take several months to be available. There are encouraging signals that regular IG will equate to HIG in terms of efficacy but at significantly lower costs. Such IG should compete with anti-Spike mAbs for pre-exposure prophylaxis in immunocompromised subjects who did not respond to vaccines, but also for PEP and early treatment in frail subjects. This is of particular relevance in light of the resistance of Omicron to mAbs and vaccine-elicited sera [49,54].

The European Commission, upon the advice of the EMA, has authorized the following medicines containing monoclonal antibodies, alone or in combination, against the spike protein of the SARS-CoV-2 virus: casirivimab–imdevimab combination for the treatment and prevention of COVID-19; regdanvimab for the treatment of COVID-19; sotrovimab for the treatment of COVID-19; and tixagevimab–cilgavimab for the pre-exposure prophylaxis of COVID-19 and in the early treatment of SARS-CoV-2 infected subjects at risk of a severe form of COVID-19 [55]. As mentioned above, several of the antibodies in clinical use might lose efficacy against the B.1.1.529 Omicron variant [56]. EMA’s Emergency Task Force (ETF) has cautioned that monoclonal antibodies currently authorized for COVID-19 are unlikely to be effective against emerging strains of SARS-CoV-2 [57].

Of note, mAbs directed against pathogens are unlikely to be used routinely due to their high cost and requirement for parenteral administration; however, they may be especially useful for certain emerging infectious diseases [58].

## 4. Arboviruses

Arboviruses (arthropod-transmitted viruses) represent an important public health challenge worldwide. At present, there are over 100 viruses classified as arboviruses, which can cause disease in humans [59]. The successful spread of these infections is determined by the complex interaction between the virus, vector, and environment. Infections occur not only in endemic areas but have often been associated with travel to endemic areas. The causes of the phenomenon are many, but climate change plays a significant role in influencing the presence, distribution, and development cycle of various arthropod species of health interest.

The main human pathogens are West Nile virus (WNV), Chikungunya virus (CHIKV), Zika virus (ZIKAV), Dengue virus (DENV), and Japanese encephalitis virus (JEV). For most of these viruses, vaccines or direct-acting antiviral drugs are not available, and the treatment regimens are mostly symptomatic, based on the clinical manifestations; therefore, passive immunotherapy could be a plausible alternative.

### 4.1. West Nile Virus (WNV)

WNV infection usually is asymptomatic but may occasionally have more severe outcomes with neurological syndromes [60]. Currently, there is no specific treatment for WNV disease, and to date, no WNV vaccines are licensed for human use. Actually, vaccines for veterinary use have been produced, and the research is focused on the development of preparations for human use [61,62]. The strategies put in place for the containment of infection are based on prevention programs that include vector control, individual protection measures to reduce exposure, and screening of blood and organ donors. In Italy, this takes place on the basis of a National Arbovirus Plan established by the Ministry of Health [63].

The available literature reports several case reports and case series on the treatment of patients with WNV disease (mainly immunocompromised with forms of meningoencephalitis) with standard and HIG, recombinant humanized monoclonal antibody, interferon, ribavirin, and corticosteroids [64,65,66,67,68,69,70]. However, in most cases, the results are inconclusive, controversial, and without statistical value. Treatment with IVIG appears to be effective in neuroinvasive manifestations [71,72].

### 4.2. Chikungunya (CHIKV)

Approximately 15% of CHIKV infections are asymptomatic [73]. The most common symptoms are fever and joint pain and can include headache, muscle pain, joint swelling, or rash. Serious cases and deaths are quite rare and almost always related to other pre-existing pathologies. There is no licensed vaccine or specific treatment for CHIKV infection. The main control measures concern individual protection and vector control [74].

Passive immunotherapy involves the administration of anti-CHIKV antibodies in high-risk individuals; it has been used successfully in experimental animal research, and there are currently clinical trials in patients susceptible to serious infections [75,76,77].

### 4.3. Zika (ZIKAV)

In 80% of cases, the infection is asymptomatic. Symptoms are flu-like and self-limiting, lasting about 2–7 days, and may be accompanied by maculopapular rash, arthralgia, myalgia, headache, and conjunctivitis. During pregnancy, ZIKAV infection can cause microcephaly and other congenital malformations in the newborn. In adults and older children, ZIKAV may be responsible for Guillain–Barré syndrome, neuropathies, and myelitis. The main control measures concern individual protection (not only from vectors but also from sexual transmission) and vector control [78].

There are currently no vaccines or preventive therapies. The efficacy of CP therapy has been experimentally evaluated on animal models; moreover, in the absence of approved vaccines or specific drugs, the engineering of neutralizing antibodies is also being evaluated [78,79,80].

### 4.4. Dengue (DENV)

Many DENV infections are asymptomatic or produce only mild forms; serious forms and even death are rarely reported [81]. The risk of severe disease is higher in people with secondary infections with different serotypes, more likely to constitute a risk factor for DENV hemorrhagic fever [82]. Prevention and control of dengue depend on vector control. Early diagnosis and access to therapies reduce the serious forms.

There is no specific treatment for DENV, and in most cases, people recover completely within two weeks. For the prevention of DENV disease, the first vaccine was produced for people who have contracted the infection at least once and who live in high-risk areas [81]. A second vaccine (live, attenuated) with broader protection (Dengue Tetravalent Vaccine) was later approved [83].

Passive immunization with serum IGs or mAbs can complement or replace vaccination for short-term dengue immune prophylaxis. However, it cannot be excluded that the use of human IG could generate an adverse event similar to a secondary infection [82,84,85,86].

### 4.5. Japanese Encephalitis Virus (JEV)

JEV is the leading cause of viral encephalitis in many Asian countries. Cases of symptomatic encephalitis are quite rare but with a mortality rate of up to 30% and 30–50% of permanent neurological or psychiatric sequelae [87]. Although there are no specific therapies for treating JEV infection, vaccines are available.

Many studies have been conducted to explore the efficacy and safety of treating severe forms of JEV infection with IVIG: the results seem promising and report good effectiveness in neutralizing JEV and reducing inflammation [88,89,90].

Other studies evaluated the efficacy of anti-JEV mAbs with high neutralizing activity in vitro on animal models. Passive immunization with anti-JEV mAb has been observed mainly in the prevention rather than in the treatment of the infection [91,92,93].

## 5. Conclusions

In recent years, due to the continuous growth of new clinical indications, extensive off-label use, and uncertainty about the duration of some treatments, a global disproportion between IG demand and availability occurred.

In order to balance the availability of the products to the patients’ needs, it is important to manage the appropriateness of the demand, providing the system with evidence-based guidelines to be applied mainly in shortage conditions. The Italian Medicines Agency (AIFA) and the Italian National Blood Centre have deemed opportune the definition and provision of general guidelines as a reference document for the appropriate use of IGs in several indications. In February 2022, the “Guidelines on the use of human immunoglobulins in case of shortages” [94] was published with the support of the main scientific societies involved in the topic. This document is based on the availability of IGs at national and regional levels, and it is proposed to adopt various specific management strategies. Furthermore, the main criteria for guaranteeing an appropriate use of IGs in shortage contexts are proposed, divided by therapeutic area and pathological condition. In addition to improving the appropriateness of demand, it is still important to guarantee supplies through collection activities and the improvement in industrial yield.

Over the years, new therapeutic strategies have been developed as alternatives to human IG, including the use of mAbs.

In 1975, the discovery of a method to produce mAbs by immortalizing B cells revolutionized antibody therapeutics [2,95]. By recombinant engineering, mAbs are produced as chimeric humanized and fully humanized. Available mAbs are directed against a large number of antigens and used for the treatment of immunologic diseases, reversal of drug effects, and cancer therapy [7]. The use of mAbs directed against infectious pathogens is an area of intensive investigation, but right now, only a few mAbs are currently employed in this field: Palivizumab, for the prevention of respiratory syncytial virus (RSV) infections, Raxibacumab and Obiltoxaximab, for the prophylaxis and treatment against anthrax toxin, Bezlotoxumab, for the prevention of *Clostridium difficile* recurrence [7,96]. Anti-SARS-CoV-2 monoclonal antibodies are an alternative option for COVID-19-specific therapy for symptomatic outpatients at risk for progression to severe disease [55,58]. A potential limitation of mAbs for the treatment of infections is the lack of knowledge of factors influencing the bioavailability of the passively infused mAbs into tissues affected [97].

It is also important to research the optimal targets for pathogens, also in relation to emerging infections, the characterization of the binding site/epitope, and the mAb specificity and selectivity to pathogens, also in order to study their real therapeutic potential.

Finally, the possibility of cross-reactivity with other human or animal cells and/or tissues cannot be excluded. These aspects, as well as the resistance to mAbs, must be the subject of study; in fact, target antigen modulation is a mechanism that determines the resistance to monoclonal antibody therapy. The potential for mAb resistance through epitope mutation should be considered and prospectively evaluated before clinical study.

While considerable efforts are being made to develop mAbs and vaccines for the treatment and prevention of infections, especially from emerging pathogens, it is still necessary to continue to ensure the self-sufficiency of plasma-derived products and equitable access to treatment. It is important to develop useful strategies for directing pharmaceutical research and development, also taking into consideration possible emergency situations from unknown pathogens.

## Figures and Tables

**Table 1:** Total demand and total standardized demand for specific immunoglobulin, expressed in International Units and International Units per capita and per 1000 population, and variations in percentage between 2017 and 2021 (adapted by the Italian National Blood Centre on data from the Traceability information flow, Ministry of Health and the Italian Medicines Agency).

	*Total Demand*					*Total Demand (Per Capita)*					Δ% 2021 vs. 2017
	2017	2018	2019	2020	2021	2017	2018	2019	2020	2021	
***CMV IG***	15,781,000	14,333,000	15,490,000	12,690,000	14,455,000	0.26	0.24	0.26	0.21	0.24	−8.40%
***Hepatitis B IG (SQ/IM use)***	64,575,800	57,886,180	75,488,160	70,073,140	62,879,640	1.07	0.96	1.25	1.17	1.06	−2.63%
***Hepatitis B IG*** ***(IV use)***	23,047,100	17,885,500	16,150,000	14,655,500	14,362,500	0.38	0.30	0.27	0.25	0.24	−37.68%
***Tetanus IG***	141,258,000	131,772,250	147,042,000	124,246,683	117,126,250	2.33	2.18	2.44	2.08	1.98	−17.08%
	***Total Demand***					Total Demand (*1000 population)					
***Rabies IG***	84,000	111,150	185,550	134,850	108,150	1.39	1.84	3.07	2.26	1.83	28.75%
***Varicella-*** ***zoster IG***	195,750	179,625	86,625	69,500	111,000	3.23	2.97	1.44	1.17	1.87	−43.30%

IG—immunoglobulin; SQ—subcutaneous; IM—intramuscular; IV—intravenous; *—per.

**Table 2:** Hyperimmune globulins for the management of infectious diseases.

Conditions	Management	Polyclonal Immunoglobulins	Hyperimmune Globulins	Comment
Tetanus	PEP with passive antibodies administration (1 dose of 250 IU TIG intramuscularly) + vaccine; for treatment of established tetanus passive antibodies (TIG, 3000–6000 IU) and vaccine, in combination with antimicrobials and muscle relaxant.	Equine antitoxin is still used in non-industrialized countries. Levels of tetanus antitoxins in polyvalent IVIG are variable.	TIG	Poor persistence of Ab levels in donors for immune plasma program [14].
*Clostridium difficile*	Antibiotics, mAbs.	IgG antitoxin is present in standard immunoglobulin preparations.	mAbs against Clostridioides difficile toxin A (actoxumab) and toxin B (bezlotoxumab) were developed.	Passive immunotherapy with intravenous immunoglobulin may be a useful addition to antibiotic therapy for severe, refractory *C difficile* colitis, but further studies are needed. Bezlotoxumab is the first mAb for secondary prevention of recurrence of *C. difficile* infection approved by the regulatory agencies in US and Europe.
Botulism	Supportive care. The role of other medical treatments is unclear. Potential medical treatments included equine serum trivalent botulism antitoxin, human-derived botulinum immune globulin intravenous (BIG-IV), plasma exchange, 3,4-diaminopyridine, and guanidine.	Equine serum antitoxins.	Human-derived botulinum immune globulin intravenous (BIG-IV) for the treatment of infant intestinal botulism.	Trivalent and heptavalent equine antitoxins are available. BIG-IV is available in the US.
Diphtheria	Vaccine, antimicrobials, antitoxin.	Equine antitoxin.	Not available.	Because of the risk of allergic reactions, equine antitoxins use is limited to diphtheria infection, not to PEP.
Cytomegalovirus (CMV)				
In SOT and HSCT	Prevention of CMV infection and disease. Treatment of established CMV disease.	IVIG, high-titer anti-CMV antibodies.	CMVIG	Further research is needed.
Congenital CMV (CCMV)				CMVIG is effective for the prevention of CCMV and mostly decreases fetal or neonatal disease.
Hepatitis A virus (HAV)	HAV vaccine is available for PrEP and PEP. IVIG for PEP (0.02 mL/kg) when the vaccine is not recommended or in an outbreak setting.	The prevalence of anti-HAV antibodies is decreasing in industrialized countries.	Preselection of donors necessary for IVIG preparation to reach satisfactory anti-HAV titers.	Depression of the immune response to inactivated HAV vaccine has been observed with concomitant administration of Ig, and a booster dose may be required sooner than when the vaccine is administered alone [15].
Hepatitis B virus (HBV)	HBIG (0.06 mL/kg) is commonly used as part of passive/active immunization in post-exposure settings, mother-to-child transmission, and post-transplant treatment of HBV. Combination of HBIG, vaccine, and antiviral drugs.		HBIG is prepared from the plasma of donors with high titers of HBV-Ab.	
Respiratory syncytial virus (RSV)	No vaccine is available, but mAbs and HIG can be considered for the prevention and treatment of RSV infection in at-risk subjects.	RSV polyclonal IVIG is no longer available.	Hyper-enriched anti-RSV Ig can be obtained from human normal plasma.	Palivizumab is the first mAbs commercially available for the prevention of an infectious disease.
Varicella-zoster virus (VZV)	PEP includes antivirals (acyclovir, valaciclovir), VZIG, IVIG, and vaccines.	The use of immune globulin for VZ infection has decreased after the varicella vaccine became part of the PEP approach in healthy adults.	ZIG and VZIG are available.	VZIG is useful in susceptible patients (no previous varicella disease or vaccination) at high risk for complications (for example, immunocompromised adults and pregnant women) when exposed to the virus.
Rabies	PEP with vaccine and concomitant HRIG (20 IU/kg).	Purified equine RIG.	HRIG is available for PEP. A recombinant human IgG anti-rabies mAbs has been developed.	In developing countries where HRIG is not available, purified equine RIG is still used.
SARS-CoV-2	Prevention and treatment of COVID-19 with drugs, vaccines, CP, Ig, HIG, and mAbs.	CP and standard Ig.	HIG, anti-spike mAbs.	Several mAbs in clinical use might lose efficacy against the Omicron variant.

PEP—post-exposure prophylaxis; PrEP—pre-exposure prophylaxis; SOT—solid organ transplantation; HSCT—hematopoietic stem-cell transplantation; TIG—tetanus immunoglobulin; mAbs—monoclonal antibodies; IgG—immunoglobulin G; IVIG—human immunoglobulin for intravenous; HIG—hyperimmune globulins; VZIG—varicella-zoster IG; ZIG—zoster immunoglobulin; HRIG—human rabies immunoglobulin; RIG—rabies immunoglobulin; CP—convalescent plasma; Ig—immunoglobulin; HBIG—hepatitis B virus immunoglobulin; CMVIG—cytomegalovirus immunoglobulin.

## Data Availability

The data provided and produced by the Italian National Blood Center are available in the ISTISAN Reports “Analysis of the demand for plasma-derived medicines in Italy.” published by the Italian National Institute of Health and available on https://www.iss.it/rapporti-istisan. Accessed on 1 June 2023.
